# Editorial: Robotic assisted laparoscopic surgery (RALS) in pediatric urology, volume II

**DOI:** 10.3389/fped.2025.1609052

**Published:** 2025-05-23

**Authors:** Alfredo Berrettini, Miguel Castellan

**Affiliations:** ^1^ Department of Pediatric Urology, Fondazione IRCCS Ca’ Granda, Ospedale Maggiore Policlinico, Milan, Italy; ^2^ Department of Pediatric Urology, Nicklaus Children’s Hospital, University of Miami, Miami, FL, United States

**Keywords:** pediatric urology, robotic surgery, laparoscopic, duplex system, testicular torsion

**Editorial on the Research Topic**
Robotic assisted laparoscopic surgery (RALS) in pediatric urology, volume II

This compilation highlights advanced research in minimally invasive surgical techniques and pediatric urology. The articles examine the detailed management of various pediatric urological conditions, significantly enhancing the field's knowledge base and encouraging innovative approaches.

Perinatal testicular torsion (PTT) is a rare form of testicular torsion that can present atypically complicating timely diagnosis and treatment (1). Huang et al. report a case involving a 50-day-old male infant, highlighting the diagnostic challenges of penile testicular transposition (PTT), particularly when it occurs with intraperitoneal cryptorchidism. They emphasize the importance of considering PTT in neonates who present with an empty scrotum and an abdominal mass. Laparoscopic exploration showed a dark brown mass approximately 3 cm in diameter in the right lower abdomen. The spermatic cord was found twisted and severely adhered. The mass was excised after vascular ligation. Pathological examination of the frozen section revealed extensive hemorrhage, necrosis, chronic and acute inflammation, and focal calcification. This case emphasizes the importance of considering PTT in neonates with scrotal asymmetry and abdominal masses. Timely imaging and surgical intervention remain critical to optimize outcomes and preserve testicular function where possible.

Minimally invasive surgeries, including laparoscopic and robotic procedures, are now frequently employed to manage ureteral anomalies associated with duplex systems. Ureteroureteroanastomosis is a technique used to relieve obstruction at the upper pole and to remove the distal remnant of the upper pole ureter while preserving the kidney's upper pole (2). Yang et al. from China, report the efficacy and safety of robot-assisted laparoscopic ipsilateral ureteroureterostomy (RAL-IUU) in treating children with duplex kidney ureteral malformations. The operation was performed on 14 children, and significant changes were observed in the anterior-posterior diameter (APD) of the upper moiety before and after surgery (23.84 ± 8.05 mm vs. 6.71 ± 2.20 mm, *P* < 0.001). Additionally, the split renal function of the upper moiety showed some improvement (12.28 ± 3.04% vs. 16.50 ± 2.75%, *P* < 0.001). During the follow-up period, none of the children experienced a urinary tract infection and all remained asymptomatic. They believed that RAL-IUU is a safe and effective treatment option for children with complete duplex kidneys and upper pole ureteral obstruction.

The Anderson-Hynes dismembered pyeloplasty is regarded as the “gold standard” surgical procedure for correcting pelvi-ureteric junction obstruction (PUJO). This procedure can be performed using open, laparoscopic, or robotic-assisted techniques (RALP) (3). In recent years, RALP has been increasingly utilized in the management of PUJO in children. Several studies have demonstrated its safety and efficacy, with success rates exceeding 90%. Vidhya et al. reported their experience over 10 years (2013–2023) involving 185 children who completed at least one year of follow-up. The average age of the group was 4.9 years (1 month to 17 years), with 25 children (13.5%) younger than 1 year old. A successful outcome (improved hydronephrosis and asymptomatic) was observed in 181 children (97.8%). Four children experienced persistent severe hydronephrosis and underwent redo stenting and/or redo pyeloplasty, resulting in a failure rate of 2.1%. This study presents the first large series of pediatric RALP from South India, demonstrating an optimal success rate even in the youngest patients, with a low complication rate. They believe that this report from their center will serve as a benchmark for other hospitals in India (and South Asia) that are embarking on a pediatric urology robotic program.

Robot-assisted laparoscopic partial nephrectomy (RALPN) is recognized as the standard treatment for small renal masses in adults, but it has been less extensively researched in pediatric patients with renal duplication anomalies (4). Batra and Dangle, offer a narrative review on the role of RALPN in managing renal duplication anomalies in pediatric populations. The authors reviewed several studies published between 2009 and 2018, comparing RALPN with open, laparoscopic, and laparoendoscopic single-site (LESS) approaches. The review highlights the advantages of robotic surgery, including reduced estimated blood loss (EBL), shorter hospital stays, lower complication rates, and improved cosmetic outcomes. Robotic procedures tend to have higher operative costs, but when factoring in shorter hospital stays and faster recoveries, overall healthcare expenses may be comparable or even reduced in the long term. The review concludes that RALPN is a safe, effective, and increasingly preferred method for managing renal duplication anomalies. However, further studies are needed to evaluate long-term outcomes, functional kidney preservation, and cost-effectiveness on a broader scale.

This Research Topic, encompassing a range of manuscripts, provides a current overview of pediatric urological management. The contributions significantly advance our understanding and treatment strategies for these conditions. We acknowledge the authors’ valuable work and anticipate that readers will find this compilation both informative and clinically relevant.

## References

[1] Monteilh C Calixte R Burjonrappa S. Controversies in the management of neonatal testicular torsion: a meta-analysis. J Pediatr Surg. (2019) 54:815819. 10.1016/j.jpedsurg.2018.07.006 30098810

[2] McLeod DJ Alpert SA Ural Z Jayanthi VR. Ureteroureterostomy irrespective of ureteral size or upper pole function: a single center experience. J Pediatr Urol. (2014) 10(4):616–9. 10.1016/j.jpurol.2014.05.003 24947344

[3] Boysen WR Gundeti MS. Robot-assisted laparoscopic pyeloplasty in the pediatric population: a review of technique, outcomes, complications, and special considerations in infants. Pediatr Surg Int. (2017) 33:925–35. 10.1007/s00383-017-4082-7 28365863

[4] Neheman A Kord E Strine AC VanderBrink BR Minevich EA DeFoor WR Pediatric partial nephrectomy for upper urinary tract duplication anomalies: a comparison between different surgical approaches and techniques. Urology. (2019) 125:196–201. 10.1016/j.urology.2018.11.026 30476504

